# The Treatment of Shoulder-boils or Cold Abscess of the Shoulder

**Published:** 1896-03

**Authors:** 


The Treatment of Shoulder-boils or Cold Abscess of the Shoulder. Article by Theodore Schmidt, D. V. S., First Clinical Assistant in the Military Veterinary Institute in Vienna.

During the school year of 1893-94 four different methods of treating shoulder-boils in horses were tried, as follows:

1. Application of moist heat with the help of a sort of collar (made to go around the neck) made of horse-blankets; fomentations, massage, and exercise.

2. Application of strong plasters, according to Hertwig's prescription.

3. Splitting of the front wall where abscess was present and the application of the sharp instrument (spoon or curette) for the partial removal of the solid walls of the abscess, followed by application of Priessnitz's poultices.

4. Extirpation of the tumor and suture, according to Bayer's method.

Two horses were treated, respectively, according to the first and second methods, six according to the third, and eleven according to the fourth.

The choice of treatment was made in the majority of cases according to the indications present as far as pedagogical considerations could be left out of account.

According to the fourth method of treatment eleven tumors were treated. Healing up took place in nine cases, as far as the drainage canal, by first intention. In two cases, however, only partly in the first manner, because in one case a part of the (button) threads had cut through, and in the second case part of them had been torn out by the horse with his teeth.

When the swelling peels off (scales off) it is only necessary, according to the statement of my chief, Dr. Bayer, to remove so much of the tumor as will leave the same cupped, so to speak. After preparing and separating the flaps (lips) of skin, the incision is to be made in a plane with the sterno-cleido-mastoideus,[1] and in following this direction out the front wall of the abscess will be reached in a great majority of cases. It will seldom be necessary to go deeper in order to open the abscess. A total extirpation of the tumor in the case of these kind of tumors, which are complicated by streptococci, and often extend beyond the jugular (furrow or channel), would be dangerous on account of the proximity of the great channel, and besides be a mistake, because a good-sized cavity, visible at a distance, would result therefrom, which would be of considerable importance for the position of the collar and harness. The solid connecting tissue on both the side-walls of the abscess-cavity, which are formed in consequence of the chronic process of inflammation, are absorbed in the course of a few weeks after the operation, when the cause is removed, so that then the contour over both points become the same.

Finally, I must mention a slight (very seldom of any extent) œdema appears on the front of the breast and toward the side-breast on the third or fourth day after the operation, but disappears again before the seventh or eighth day. The infiltration of the skin ceases in a few days after the removal of the threads, and the skin becomes again pliant and elastic.

In the case of a cold abscess of no long standing and not very large, if the swelling has only a moderately firm consistency and involves only the cellular tissue of the under skin, the first

[1] Part of mastoido-humeralis of. Chauveau, Fleming's translation.

method of treatment with moist heat will usually prove effective. A strong plaster can be applied here also, but the first method has this advantage over the second, that the condition of the swelling can be observed every day.

The application of a severe plaster or blister is good when one does not intend to visit the patient again for several days. But the adherence of the plaster to the semispherical swelling and the tearing off of large pieces of the same after exudation has set in is a great disadvantage.

If the diagnosis is an abscess involving the cellular tissue of the under skin, so that a large part of the swelling shows an inflammatory œdema, then the third method, with splitting of the abscess, is to be recommended.

In every kind of tumors (joint-boils), which consist of a very tough connective tissue, which has its seat in the subcuticle, or which rests on a hyperplasia of the tissues in the sterno-cleido-mastoideus, and is supported through the process of local suppuration in the muscle, the method of Bayer, with peeling off of the swelling, and the suture has the preference.

While many complain of the unsatisfactory results of this method and question the value of the same, I must answer that conditions are the same as in the application of Lister's method, and it must be determined, in the first place, whether the trouble does not lie in the carrying out of the treatment.

Even in those cases where a breaking of the intra- and sub-muscular abscess into the subcutaneous cellular tissue has taken place and an abscess has been formed here, the peeling (scaling) off of the indurated masses, and the application of the suture is, according to Bayer, to be preferred to treatment as an open wound, because healing up follows much more quickly, and a great deal of trouble is spared incident to the treatment of open wounds.—*Thierärzt Centralblatt*, December 1, 1895.

